# Supplementary material for: New Axes of Interaction in Circ_0079593/miR-516b-5p Network in Melanoma Metastasis Cell Lines
Source: Genes (Basel). 2024 Dec 21;15(12):1647. doi: 10.3390/genes15121647 (PMC11675925; doi:10.3390/genes15121647)
Supplement: Supplementary file 1 [file genes-15-01647-s001.zip › genes-3370266-supplementary.pdf]

***Table S1**: Screenshot of potential miRNA-circRNA interactions table generated by circInteractome after selecting hsa_circ_0079593*

<table>
<tr><th>CircRNA
Mirbase ID</th><th>CircRNA (Top) - miRNA (Bottom) pairing</th><th>Site Type</th><th>context+ score
percentile</th></tr>
<tr><td>hsa_circ_0079593 (5′ ... 3′)

hsa-miR-1292 (3′ ... 5′)</td><td>GAACGCCUUGGGUCUGUUCCCAC
||||||
GUCGCAGACGGCCUUGGGCAAGGGU</td><td>7mer-1a</td><td>84</td></tr>
<tr><td>hsa_circ_0079593 (5′ ... 3′)

hsa-miR-1299 (3′ ... 5′)</td><td>AUUGACGCUGUAUAAUCCAGAAC
||||||
AGGGAGUGUGUCUUAAGGUCUU</td><td>7mer-1a</td><td>88</td></tr>
<tr><td>hsa_circ_0079593 (5′ ... 3′)

hsa-miR-182 (3′ ... 5′)</td><td>CAAUGUUGAGACAUGUGCCAAAG
||||||
UCACACUCAAGAUGGUAACGGUUU</td><td>7mer-1a</td><td>85</td></tr>
<tr><td>hsa_circ_0079593 (5′ ... 3′)

hsa-miR-324-5p (3′ ... 5′)</td><td>UUCCCACCCACUUCAGGGAUGCC
|||||||
UGUGGUUACGGGAUCCCCUACGC</td><td>7mer-m8</td><td>91</td></tr>
<tr><td>hsa_circ_0079593 (5′ ... 3′)

hsa-miR-433 (3′ ... 5′)</td><td>AAAGCUGAGGAGGAGAUCAUGAA
|||||| |||||||
UGUGGCUCCUCGGGUAGUACUA</td><td>8mer-1a</td><td>99</td></tr>
<tr><td>hsa_circ_0079593 (5′ ... 3′)

hsa-miR-516b (3′ ... 5′)</td><td>AUUGACGCUGUAUAAUCCAGAAC
||||||
UUUCACGAAGAAUGGAGGUCUA</td><td>7mer-1a</td><td>86</td></tr>
<tr><td>hsa_circ_0079593 (5′ ... 3′)

hsa-miR-633 (3′ ... 5′)</td><td>UAUAAUCCAGAACGCACUAUUAC
|||||||
AAAUAACACCAUCUAUGAUAAUC</td><td>7mer-m8</td><td>90</td></tr>
<tr><td>hsa_circ_0079593 (5′ ... 3′)

hsa-miR-636 (3′ ... 5′)</td><td>GCUUCUAUGAAUCUUCAAGCACA
|||||||
ACGCCCGCCCUGCUCGUUCGUGU</td><td>8mer-1a</td><td>99</td></tr>
<tr><td>hsa_circ_0079593 (5′ ... 3′)

hsa-miR-665 (3′ ... 5′)</td><td>UCAAGCACAUUUAAUUCCUGGAU
||||||
UCCCCGGAGUCGGAGGACCA</td><td>7mer-1a</td><td>93</td></tr>
</table>
